# Increased ribosomal protein levels and protein synthesis in the striatal synaptosome of Shank3-overexpressing transgenic mice

**DOI:** 10.1186/s13041-021-00756-z

**Published:** 2021-02-23

**Authors:** Chunmei Jin, Yeunkum Lee, Hyojin Kang, Kwon Jeong, Joori Park, Yinhua Zhang, Hyae Rim Kang, Ruiying Ma, Hyunyoung Seong, Yoonhee Kim, Hosung Jung, Jin Young Kim, Yoon Ki Kim, Kihoon Han

**Affiliations:** 1 Department of Neuroscience, College of Medicine, Korea University, 73, Goryeodae-ro, Seongbuk-gu, Seoul, 02841 Republic of Korea; 2 Department of Biomedical Sciences, College of Medicine, Korea University, Seoul, Republic of Korea; 3 Division of National Supercomputing, Korea Institute of Science and Technology Information (KISTI), Daejeon, Republic of Korea; 4 Creative Research Initiatives Center for Molecular Biology of Translation, Korea University, Seoul, Republic of Korea; 5 Division of Life Sciences, Korea University, Seoul, 02841 Republic of Korea; 6 Brain Korea 21 PLUS Project for Medical Science, Yonsei University College of Medicine, Seoul, Republic of Korea; 7 Department of Anatomy, Brain Research Institute, Severance Biomedical Science Institute, Yonsei University College of Medicine, Seoul, Republic of Korea; 8 Research Center for Bioconvergence Analysis, Korea Basic Science Institute (KBSI), Ochang, Republic of Korea

**Keywords:** Shank3, Ribosomal protein, Protein synthesis, Striatum, Synaptosome

## Abstract

The SH3 and multiple ankyrin repeat domains 3 (Shank3) protein is a core organizer of the macromolecular complex in excitatory postsynapses, and its defects cause numerous synaptopathies, including autism spectrum disorders. Although the function of Shank3 as a postsynaptic scaffold is adequately established, other potential mechanisms through which Shank3 broadly modulates the postsynaptic proteome remain relatively unexplored. In our previous quantitative proteomic analysis, six up-regulated ribosomal proteins were identified in the striatal synaptosome of Shank3-overexpressing transgenic (TG) mice. In the present study, we validated the increased levels of RPLP1 and RPL36A in synaptosome, but not in whole lysate, of the TG striatum. Moreover, protein synthesis and extracellular signaling-regulated kinase (ERK) activity were enhanced in the TG striatal synaptosome. To understand the potential contribution of increased protein synthesis to the proteomic change in the TG striatal synaptosome, we performed RNA-sequencing analyses on both whole synaptosomal and synaptic polysome-enriched fractions. Comparative analyses showed a positive correlation only between the polysome-associated transcriptome and up-regulated proteome in the TG striatal synaptosome. Our findings suggest a novel mechanism through which Shank3 may remodel the postsynaptic proteome by regulating synaptic protein synthesis, whose dysfunction can be implicated in *SHANK3*-associated synaptopathies.

## Main text

Shank3 protein is a core organizer of the macromolecular complex in the postsynaptic density (PSD) of neuronal excitatory synapses [1]. Consistent with its critical roles in proper synaptic development and function, variants of the *SHANK3* gene have been causally associated with numerous synaptopathies [2]. Shank3 interacts with many other synaptic proteins through its multiple protein–protein interaction domains, thereby regulating their synaptic localization and stability [3]. This “scaffolding” function is a well-established mechanism underlying the organization of the PSD complex by Shank3. However, considering the highly dynamic regulation of synaptic proteins, including local synthesis and turnover [4], there can be additional, yet unexplored, mechanisms through which Shank3 orchestrates the postsynaptic proteome.

**Fig. 1:**
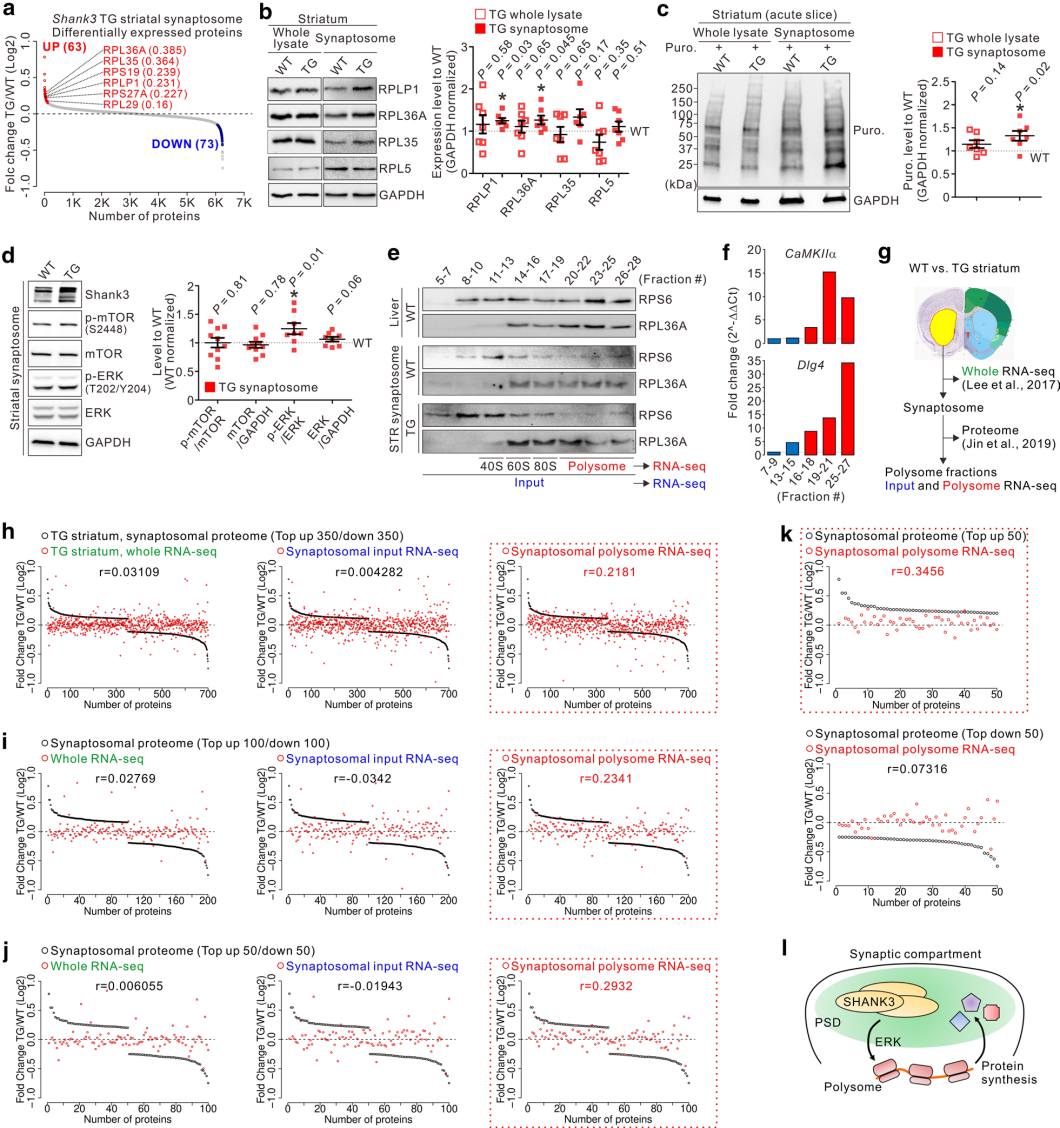
Increased ribosomal protein levels, protein synthesis, and ERK activity in the striatal synaptosome of Shank3-overexpressing transgenic (TG) mice compared with wild-type (WT) mice. **a** The graph shows relative protein abundance between the TG and WT striatal synaptosomes. The six ribosomal proteins (RPs) up-regulated in TG mice and their fold-change values are indicated. **b** Western blot images and graph showing the expression levels of RPs in whole lysate and synaptosome of the TG striatum compared with the WT striatum (n = 6–7 mice per genotype). **c** Western blot images and graph showing the levels of puromycin (Puro.) labeling in whole lysate and synaptosome of the WT and TG striatal slices (n = 7 mice per genotype). **d** Western blot images and graph showing the levels of total and phosphorylated mTOR and ERK in the striatal synaptosome of WT and TG mice (n = 8–10 mice per genotype). **e** Western blot images showing the distribution of the RPs, RPS6 and RPL36A, in different sucrose density-gradient fractions of the WT and TG striatal synaptosome. Samples from WT liver were loaded as a positive control for sucrose density-gradient fractions. **f** Graph showing the results of qRT-PCR validation for the enrichment of *CaMKIIa* and *Dlg4* mRNAs in polysome-enriched fractions of the striatal synaptosome. **g** Schematic diagram showing the multi-omics datasets of the WT and TG striata. **h**–**j** Graphs showing the comparisons of fold-change values between the proteomic change and each of the transcriptomic changes in the TG striatum. **k** Graphs showing the comparisons of fold-change values between the top 50 up-regulated proteins and synaptic polysome-associated transcripts (upper panel), as well as between the top 50 down-regulated proteins and synaptic polysome-associated transcripts (lower panel) in the TG striatum. **l** Schematic diagram showing a hypothesis that Shank3, via the ERK pathway, regulates synaptic protein synthesis, which provides proteins to the postsynaptic density (PSD). Data are presented as the mean ± SEM. *P < 0.05 (unpaired two-tailed Student’s *t*-test). All raw image and quantification data for Western blotting is provided in Additional files 3 and 4

By applying a quantitative proteomic analysis, we recently identified several differentially expressed (63 up-regulated/73 down-regulated) proteins in the striatal synaptosome of *Shank3* TG mice compared with wild-type (WT) mice [5]. Unexpectedly, we found that six ribosomal proteins (RPs) were included in the up-regulated proteins of the TG striatal synaptosome (Fig. 1a). Consistently, bioinformatic analyses revealed that several ribosome-related terms were significantly represented by the up-regulated proteins (Additional file 1: Figs. S1 and S2). Furthermore, Western blot analysis validated the increased levels of RPLP1 and RPL36A in synaptosome of the TG striatum compared with the WT striatum (Fig. 1b). Notably, in whole lysate, those protein levels were comparable between the TG and WT striata, suggesting that an increase in RP levels was specific to synaptosome.

Based on the above results, we measured the efficiency of global protein synthesis or mRNA translation in WT and TG striata, by using a puromycin incorporation assay (Additional File 1: materials and methods). We used acute striatal slices from WT and TG mice and confirmed that proteins in both whole lysate and synaptosome could be labeled by incubating the slices with puromycin (Additional File 1: Fig. S3). Labeling was suppressed by pretreatment with the protein synthesis inhibitor, cycloheximide, suggesting that puromycin signals represent nascent polypeptides. When we measured the intensity of puromycin labeling, it was significantly increased in synaptosome, but not in whole lysate, of the TG striatum compared with the WT striatum (Fig. 1c). This result suggests that, similar to the increase in RP levels, protein synthesis was enhanced only in synaptosome of the TG striatum.

The mechanistic target of rapamycin (mTOR) and mitogen-activated protein kinase (MAPK)/ERK pathways are key regulators of synaptic protein synthesis [6]. We previously showed decreased mTOR complex 1 (mTORC1) activity in whole lysate of the TG striatum compared with the WT striatum [7, 8]. However, since increased RP levels and protein synthesis in the TG striatum were observed specifically in synaptosome, we measured the activities of mTORC1 and ERK in the striatal synaptosome of WT and TG mice. Unlike in whole lysate [7], mTORC1 activity was normal, but ERK activity was significantly increased in the TG striatal synaptosome (Fig. 1d). The total levels of mTOR and ERK proteins were comparable between the WT and TG striata.

Intriguingly, when we compared the list of 63 up-regulated proteins in the TG striatal synaptosome [5] with that of the recently reported comprehensive Shank3 interactome (793 proteins) [9], only eight proteins, including Shank3 itself, were shared by both lists (Additional File 2: Table S1). This unexpectedly low percentage of Shank3-interacting proteins in the up-regulated proteome (12.7%) suggests that interaction-mediated recruitment by overexpressed Shank3 (i.e., the scaffolding function of Shank3) may only partially contribute to the proteomic change in the TG striatal synaptosome. Therefore, we investigated whether increased protein synthesis may be associated with proteomic change in the TG striatal synaptosome. To examine this, we performed sucrose density-gradient fractionation with the striatal synaptosome of WT and TG mice and then purified RNAs from whole synaptosomal (referred to as “input”) or only polysome-enriched (“polysome”) fractions (Fig. 1e). We validated the relative enrichment of mRNAs in the polysome fractions by measuring the amounts of two well-known synaptic mRNAs (*CaMKIIa* and *Dlg4*) [10] (Fig. 1f). The purified synaptosomal input and polysome RNAs from three pairs of WT and TG striatal samples were further processed for next-generation RNA-sequencing (RNA-seq) (Additional File 2: Table S2).

We previously performed whole tissue, as opposed to synaptosome-enriched, RNA-seq on the WT and TG striata [7]. Therefore, using the obtained multi-omics datasets, we could compare correlations between proteomic change and three different transcriptomic changes in the TG striatum (Fig. 1g). Using proteomic change as a standard, we calculated its correlations with each of the three RNA-seq datasets by matching their fold-change values for each protein. Notably, as we narrowed down the list of proteins, from the top 700 (350 up-regulated/350 down-regulated) to the top 100, based on their fold-change values, mRNA level change in polysome, but not in whole tissue and input, showed a gradual increase in the correlation coefficient to the proteomic change (Fig. 1h–j). In addition, with any group of protein lists, polysome showed a markedly higher correlation coefficient than the whole tissue and input coefficients. Moreover, when we separately analyzed the top 50 up-regulated and 50 down-regulated proteins, only the up-regulated proteins showed a further increase in correlation coefficient with respect to the polysome data (Fig. 1k). These results suggest a positive correlation between polysome-associated transcriptomic and up-regulated proteomic changes in the TG striatal synaptosome.

Here, we showed increased RP levels and protein synthesis in the striatal synaptosome of Shank3-overexpressing mice. Elevated ERK activity, which directly interacts with Shank3 [11], may mediate the process [6, 12]. Based on our comparative analyses, we cautiously speculate that increased protein synthesis from the synaptic polysome-associated transcripts in the TG striatum may contribute to the remodeling of its postsynaptic proteome (Fig. 1l). The correlation between them might be underestimated because our proteomic data is a “snapshot” which is a net outcome of protein synthesis and degradation. Therefore, direct identification of the locally synthesized proteome [13] in the TG striatal synaptosome will be an important direction for future studies.

Among the up-regulated proteins in the TG striatum, dopamine D1 receptor (DRD1) and its downstream target, dopamine and cAMP regulated phosphoprotein 32 kDa (DARPP-32), showed up-regulated polysome-associated mRNA levels in the TG striatal synaptosome (Additional File 2: Table S3). Considering mania-like behaviors, such as hyperlocomotion and amphetamine hypersensitivity, observed in *Shank3* TG mice [3, 14], increased local synthesis of DRD1 and DARPP-32, if validated, may possibly contribute to the synaptic and behavioral changes in TG mice.

In conclusion, our study provides evidence suggesting the convergence of synaptic scaffolds and protein synthesis, where abnormalities are considered major pathogenic mechanisms underlying numerous synaptopathies [15, 16].

## Supplementary Information

**Additional file 1: Fig. S1** Gene ontology analysis for the up-regulated (A) and down-regulated proteins (B) in the striatal synaptosome of *Shank3* TG mice. **Fig. S2** Gene set enrichment analysis (GSEA) for the proteomic change in the striatal synaptosome of *Shank3* TG mice. **Fig. S3** Puromycin (Puro.) labeling of nascent polypeptides in acute slices of the mouse striatum. CHX, cycloheximide. **Materials and methods**

**Additional file 2: Table S1.** List of 63 up-regulated proteins in the striatal synaptosome of Shank3 TG mice. **Table S2.** Summary of RNA-seq mapping results. **Table S3.** List of proteins identified from the quantitative proteomic analysis in the striatal synaptosome of Shank3 TG mice, and their fold changes values for each data set are shown.

**Additional file 3.** All raw images for entire membranes of Western blotting.

**Additional file 4.** All raw numerical data for image quantification.

## Data Availability

The datasets used and analyzed in the current study are available from the corresponding author on reasonable request.

## Abbreviations

DARPP-32: Dopamine and cAMP regulated phosphoprotein 32 kDa
DRD1: Dopamine D1 receptor
ERK: Extracellular signal-regulated kinase
MAPK: Mitogen-activated protein kinase
mTOR: Mechanistic target of rapamycin
PSD: Postsynaptic density
RNA-seq: RNA-sequencing
RP: Ribosomal protein
Shank3: SH3 and multiple ankyrin repeat domains 3
TG: Transgenic
WT: Wild-type
