# Supplementary material for: Increased ribosomal protein levels and protein synthesis in the striatal synaptosome of Shank3-overexpressing transgenic mice
Source: Mol Brain. 2021 Feb 23;14:39. doi: 10.1186/s13041-021-00756-z (PMC7903774; doi:10.1186/s13041-021-00756-z)
Supplement: Supplementary file 1 — Additional file 1: Fig. S1 Gene ontology analysis for the up-regulated (A) and down-regulated proteins (B) in the striatal synaptosome of Shank3 TG mice. Fig. S2 Gene set enrichment analysis (GSEA) for the proteomic change in the striatal synaptosome of Shank3 TG mice. Fig. S3 Puromycin (Puro.) labeling of nascent polypeptides in acute slices of the mouse striatum. CHX, cycloheximide. Materials and methods [file 13041_2021_756_MOESM1_ESM.docx]

**Additional File 1.**

**Increased ribosomal protein levels and protein synthesis in the striatal synaptosome of Shank3-overexpressing transgenic mice**

Chunmei Jin, Yeunkum Lee, Hyojin Kang, Kwon Jeong, Joori Park, Yinhua Zhang, Hyae Rim Kang, Ruiying Ma, Hyunyoung Seong, Yoonhee Kim, Hosung Jung, Jin Young Kim, Yoon Ki Kim, Kihoon Han

**Additional Figures**

**
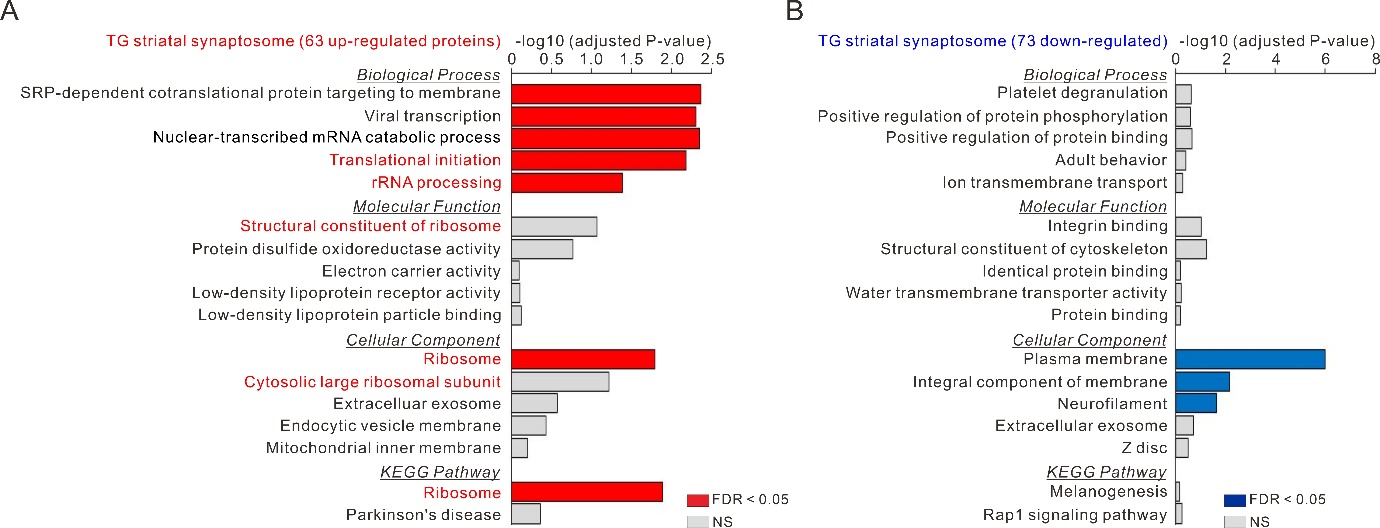
**

**Fig. S1** Gene ontology analysis for the up-regulated (A) and down-regulated proteins (B) in the striatal synaptosome of *Shank3* TG mice.

**
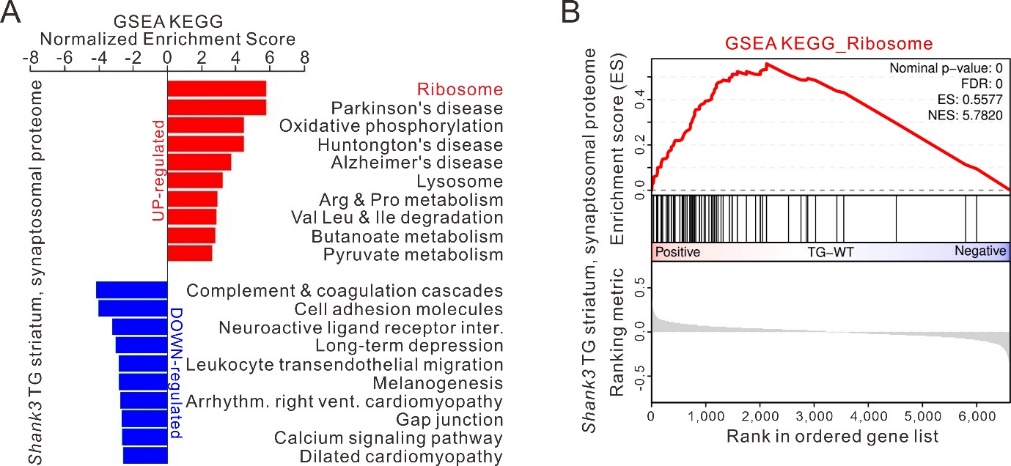
**

**Fig. S2** Gene set enrichment analysis (GSEA) for the proteomic change in the striatal synaptosome of *Shank3* TG mice. **(A)** Graph showing the top 10 enriched terms for the up-regulated and down-regulated proteins in the TG striatal synaptosome. **(B)** The enrichment plot of the proteomic change in TG mice on the ribosome gene set.

**
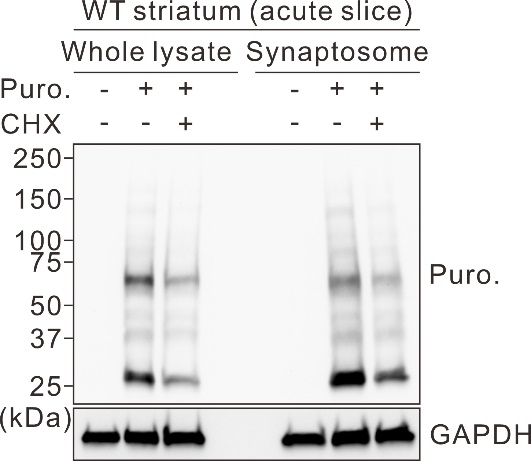
**

**Fig. S3** Puromycin (Puro.) labeling of nascent polypeptides in acute slices of the mouse striatum. CHX, cycloheximide.

**Materials and Methods**

**Mice**

The enhanced green fluorescent protein (EGFP)-Shank3-overexpressing transgenic mice used in this study have been previously described [1, 2]. The mice were bred and maintained in a C57BL/6J (Japan SLC, Inc., Shizuoka, Japan, RRID:MGI:5488963) background according to the Korea University College of Medicine Research Requirements, and all experimental procedures were approved by the Committee on Animal Research at the Korea University College of Medicine (KOREA-2018-0003). The mice were fed and had access to water ad libitum and were group housed (4–6 mice per cage) under a 12-h light-dark cycle at 18–25 °C. For all experiments, only adult (10–12-week old) male mice were used, and WT control refers to the WT littermates of the TG mice.

**Western blotting**

The protein concentration was measured using the Bradford Protein Assay (Bio-Rad, #500-0006). The lysate was heated in 1X NuPAGE LDS sample buffer (Thermo Fisher Scientific, #NP0007) containing 1X NuPAGE reducing agent (Thermo Fisher Scientific, #NP0004). From each sample, 10–20 μg of protein was loaded into 10% SDS-polyacrylamide gel or 4–15% Mini-PROTEAN TGX™ Precast Protein Gels (Bio-Rad, #4561084) for western blotting. The proteins were transferred to the nitrocellulose membrane (GE Healthcare, #10600001) or to the PVDF membrane (Millipore, #IPVH00010, for puromycin blot only). The primary antibodies used for western blot analysis were Shank3 (Santa Cruz, sc-30193, 1:1000), RPLP1(Sigma, #HPA003368, 1:500), RPL36A (Abnova, #H00006173-M02, 1:1000), RPL35 (Abcam, #Ab190162, 1:1000), RPL5 (Abcam, ab137617, 1:1000), RPS6 (Cell Signaling, #2217, 1:1000), puromycin (Millipore, #MABE343, 1:2000), phospho-mTOR (S2448) (Cell Signaling, #2971, 1:1000), mTOR (Cell Signaling, #2983, 1:1000), phospho-ERK (T202/Y204) (Cell Signaling, #4370, 1:1000), ERK (Cell Signaling, #4695P, 1:1000), and GAPDH (Cell Signaling, #2118, 1:3000). The RPL36A, RPL35, and RPLP1 were the first, second, and fourth up-regulated RPs in our quantitative proteomic analysis. The RPL5 was a negative control (i.e., RPL5 was not significantly changed in our proteomic analysis). The RPS6 and RPL36A were routinely analyzed for the sucrose density-gradient polysome fractionation.

**Puromycin incorporation assay**

Puromycin incorporation assay was performed as previously described [3] with slight modifications. Briefly, acute striatal slices (200 μm thickness) prepared using vibratome (Leica, VT1000S) were incubated in oxygenated (95% O_2_, 5% CO_2_) artificial cerebrospinal fluid (ACSF) (Ecocyte Bioscience, LRE-S-LSG-1000-2) for 20 min at 32℃ for recovery. Then slices were transferred to oxygenated ACSF containing 100 μg/mL puromycin (Sigma, P8833) and incubated for 20 min at 32℃. To block protein synthesis, slices were pre-treated with 100 μg/mL cycloheximide (Sigma, C7698) for 20 min. After puromycin labeling, the striatum was quickly cut out from each slice, frozen with liquid nitrogen, and stored at -80℃ until further processed.

**Synaptosome preparation**

The whole lysate and synaptosome were prepared as previously described [4]. Briefly, for whole lysate preparation, the frozen striatal tissues were homogenized in buffered sucrose (0.32 M sucrose, 4 mM HEPES, 1 mM MgCl_2_, 0.5 mM CaCl_2_, pH 7.3) with freshly added protease and phosphatase inhibitors (Sigma-Aldrich, St. Louis, MO, USA, #04906837001 and # 05892970001, respectively). 100 μg/mL cycloheximide and 0.2 U/μL RNase inhibitor (Thermo Fisher Scientific, #EO0381) were added to the homogenization buffer for puromycin-labeled samples. For synaptosome preparation, whole homogenate was further centrifuged at 900 g for 10 min at 4℃, and the resulting supernatant was centrifuged at 12,000 g for 15 min at 4℃, then, the pellet was resuspended in buffered sucrose and centrifuged at 13,000 g for 15 min (the resulting pellet is synaptosome).

**Sucrose density-gradient polysome fractionation**

The synaptosomal pellet (described above) was resuspended in polysome lysis buffer (50 mM MOPS, 15 mM MgCl_2,_ 150 mM NaCl, 100 μg/mL cycloheximide, 0.5% Triton X-100, 0.5% sodium deoxycholate, 1 mg/mL heparin, 0.2 U/μL RNase inhibitor, 2 mM PMSF, 1 mM benzamidine) and gently rotated for 10 min at 4℃. The lysate was centrifuged at 13,000 g for 10 min and the resulting supernatant was processed for sucrose density-gradient fractionation. The supernatant was loaded onto the top of a 10–50% (w/v) sucrose gradient, followed by ultracentrifugation at 36,000 rpm for 2 h at 4°C (Optima XE-90 Ultracentrifuge, Beckman Coulter). Each fraction was collected (a Foxy Jr Density Gradient System, Teledyne ISCO) and subjected to RNA purification.

**RNA purification from sucrose density-gradient fractions**

60 μL of fraction samples were mixed with 140 μL DEPC-DW (Invitrogen, #750023), 500 μL trizol (Ambion, #15596026), and 100 μL of chloroform (WAKO, #038-02606), vortexed, and incubated for 15 min in ice. The mixture was centrifuged at 12,000 g for 10 min, and the top aqueous layer was transferred into a new tube. 2.5 X volume of 100% ethanol (Merck, #100983.1011) and 100 μL of 8 M lithium chloride (Sigma-Aldrich #L7026) were added to the tube and the mixture was incubated overnight at -20℃. The sample was centrifuged at 12,000 g for 10 min at 4℃, and the resulting RNA pellet was washed with 70% ethanol and stored in 70% ethanol at -80℃ until further processed for RNA-seq.

**Real-time quantitative reverse transcription PCR (qRT-PCR)**

cDNA was synthesized using the iScript™ cDNA Synthesis Kit (Bio-Rad, #BR170-8891). Target mRNAs were detected and quantified by a real-time PCR instrument (CFX96 Touch, Bio-Rad) using SYBR Green Master Mix (Bio-Rad, #BT170-8884AP). The results were analyzed using the comparative Ct method and were normalized against the levels of the artificially added gene Fluc. The primer sequences for real-time PCR are as follows:

*Fluc* forward 5’-CAACACCCCAACATCTTCG-3’

reverse 5’-CTTTCCGCCCTTCTTGGCC-3’

*CaMKII* forward 5’-ACCTGCACCCGATTCACAG-3’,

reverse 5’-TGGCAGCATACTCCTGACCA-3’

*Dlg4* forward 5’-TGAGATCAGTCATAGCAGCTACT-3’,

reverse 5’-CTTCCTCCCCTAGCAGGTCC-3’

**RNA-Sequencing**

RNA extraction, library preparation, cluster generation, and sequencing were performed by Macrogen Inc. (Seoul, Korea). RNA samples for sequencing were prepared using a TruSeq Stranded Total RNA LT Sample Prep Kit (Illumina) according to the manufacturer’s instructions. An Illumina’s platform was used for sequencing to generate 101-bp paired-end reads. Raw data were submitted to the GEO (Gene Expression Omnibus) repository under accession number GSE163528. Transcript abundance was estimated with Salmon (v0.11.2) [5] in Quasi-mapping-based mode onto the Mus musculus genome (GRCm38) with GC bias correction (--gcBias). Quantified gene-level abundance data was imported to R (v.3.4.4) with the tximport (v1.6.0) [6] package and differential gene expression analysis was carried out using R/Bioconductor DEseq2 (v1.19.11) [7]. Gene Set Enrichment Analysis (GSEA) (http://software.broadinstitute.org/gsea) [8] was used to determine whether ribosome-related terms were significantly represented in the striatal synaptosome of *Shank3* TG mice with gene set collections downloaded from Molecular Signature Database (MSigDB) v7.0.

**Correlation calculation**

The correlations between proteomic changes and their matching transcriptomic changes from RNA-seq data were estimated by Pearson's correlation coefficient, which measures a linear dependence between two variables. The Pearson coefficient r was calculated using *cor* function implemented in R's stats package.

**Quantification and statistical analysis**

Values from at least three independent experiments with biological replicates were used for quantification and statistical analyses. All analyses were carried out in a blinded manner. *P* values were calculated by two-tailed Student’s t-tests using GraphPad Prism 5 software. All data are presented as mean ± standard error of the mean (SEM). **P* < 0.05.

**References**

1. Han K, Holder JL, Jr., Schaaf CP, Lu H, Chen H, Kang H et al. SHANK3 overexpression causes manic-like behaviour with unique pharmacogenetic properties. Nature. 2013;503(7474):72-7. doi:10.1038/nature12630.

2. Jin C, Kim S, Kang H, Yun KN, Lee Y, Zhang Y et al. Shank3 regulates striatal synaptic abundance of Cyld, a deubiquitinase specific for Lys63-linked polyubiquitin chains. Journal of neurochemistry. 2019;150(6):776-86. doi:10.1111/jnc.14796.

3. Zhu PJ, Khatiwada S, Cui Y, Reineke LC, Dooling SW, Kim JJ et al. Activation of the ISR mediates the behavioral and neurophysiological abnormalities in Down syndrome. Science. 2019;366(6467):843-9. doi:10.1126/science.aaw5185.

4. Han K, Kim MH, Seeburg D, Seo J, Verpelli C, Han S et al. Regulated RalBP1 binding to RalA and PSD-95 controls AMPA receptor endocytosis and LTD. PLoS biology. 2009;7(9):e1000187. doi:10.1371/journal.pbio.1000187.

5. Patro R, Duggal G, Love MI, Irizarry RA, Kingsford C. Salmon provides fast and bias-aware quantification of transcript expression. Nature methods. 2017;14(4):417-9. doi:10.1038/nmeth.4197.

6. Soneson C, Love MI, Robinson MD. Differential analyses for RNA-seq: transcript-level estimates improve gene-level inferences. F1000Research. 2015;4:1521. doi:10.12688/f1000research.7563.2.

7. Love MI, Huber W, Anders S. Moderated estimation of fold change and dispersion for RNA-seq data with DESeq2. Genome biology. 2014;15(12):550. doi:10.1186/s13059-014-0550-8.

8. Subramanian A, Tamayo P, Mootha VK, Mukherjee S, Ebert BL, Gillette MA et al. Gene set enrichment analysis: a knowledge-based approach for interpreting genome-wide expression profiles. Proceedings of the National Academy of Sciences of the United States of America. 2005;102(43):15545-50. doi:10.1073/pnas.0506580102.
