# Supplementary figures and images for: Increased ribosomal protein levels and protein synthesis in the striatal synaptosome of Shank3-overexpressing transgenic mice
Source: Mol Brain. 2021 Feb 23;14:39. doi: 10.1186/s13041-021-00756-z (PMC7903774; doi:10.1186/s13041-021-00756-z)

**Figure 1B.**

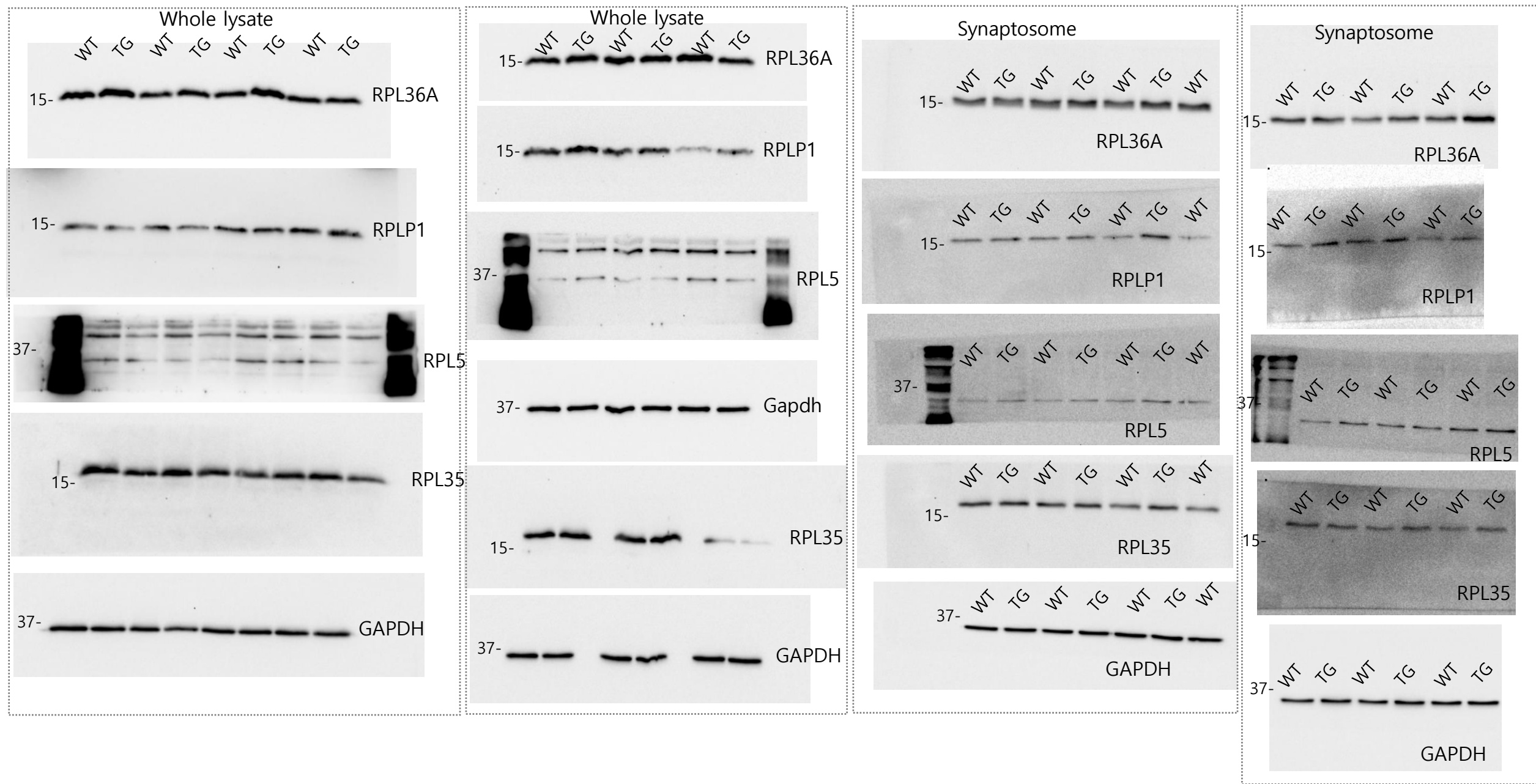

**Figure 1C.**

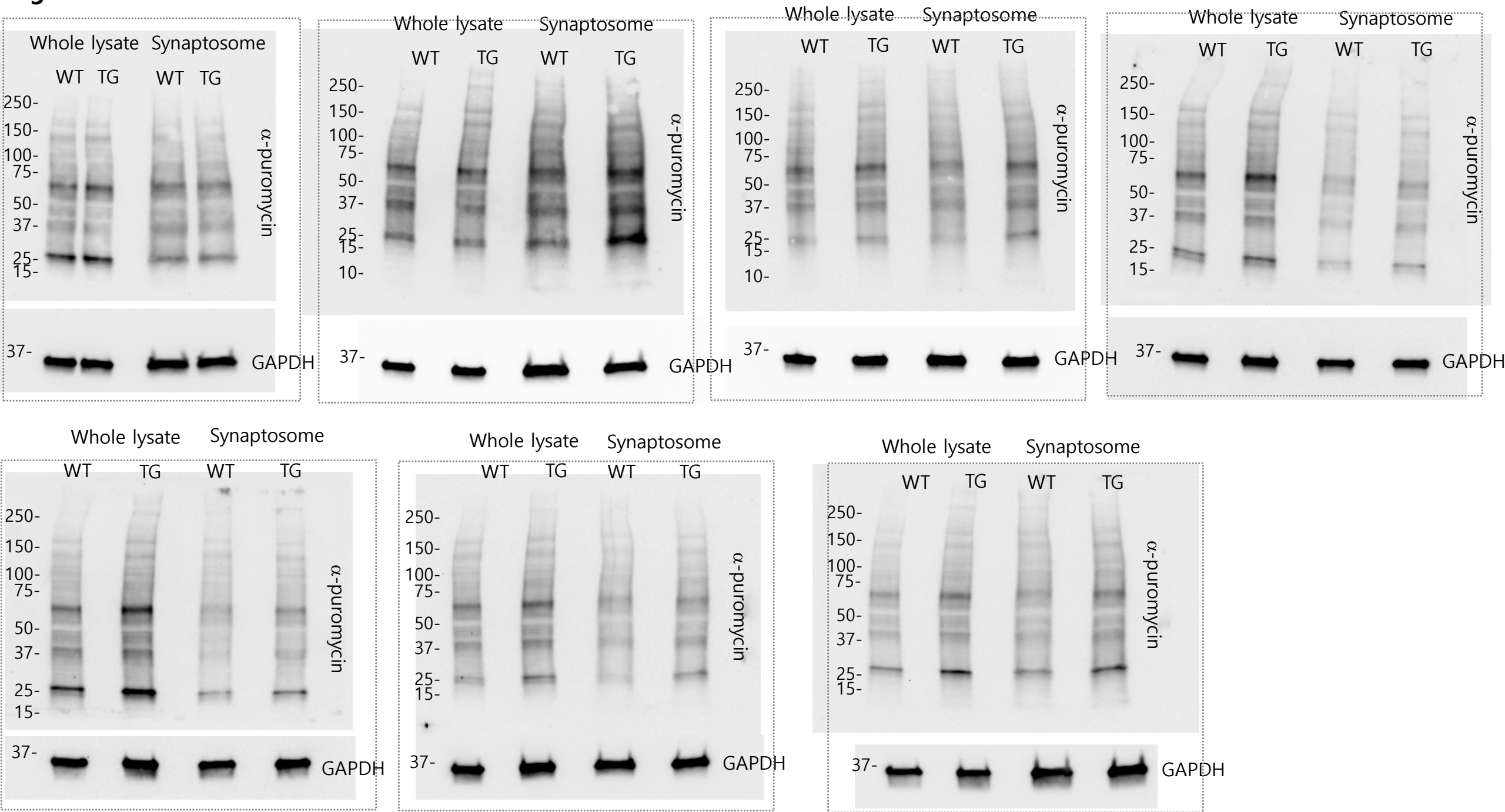

**Figure 1D.**

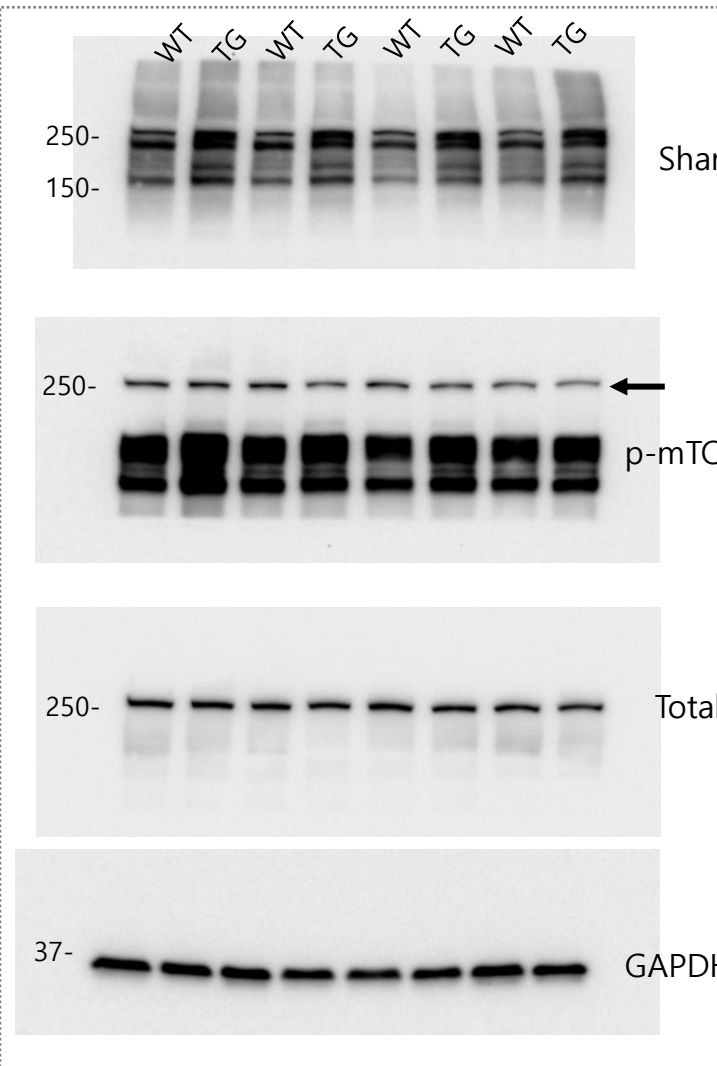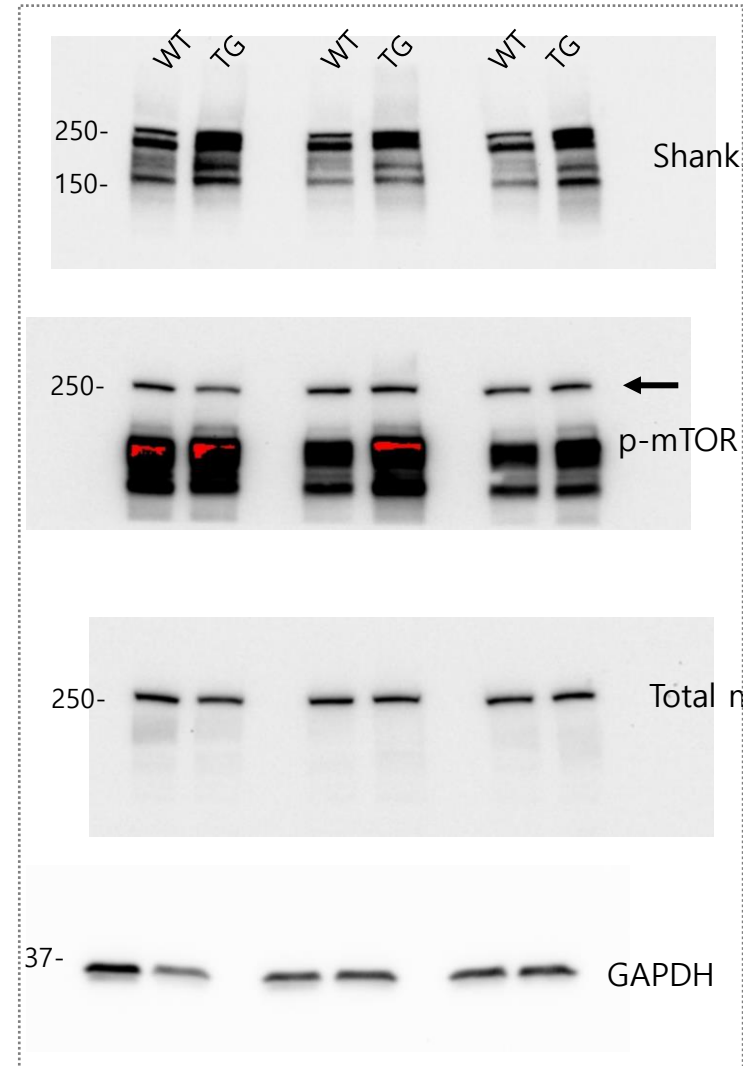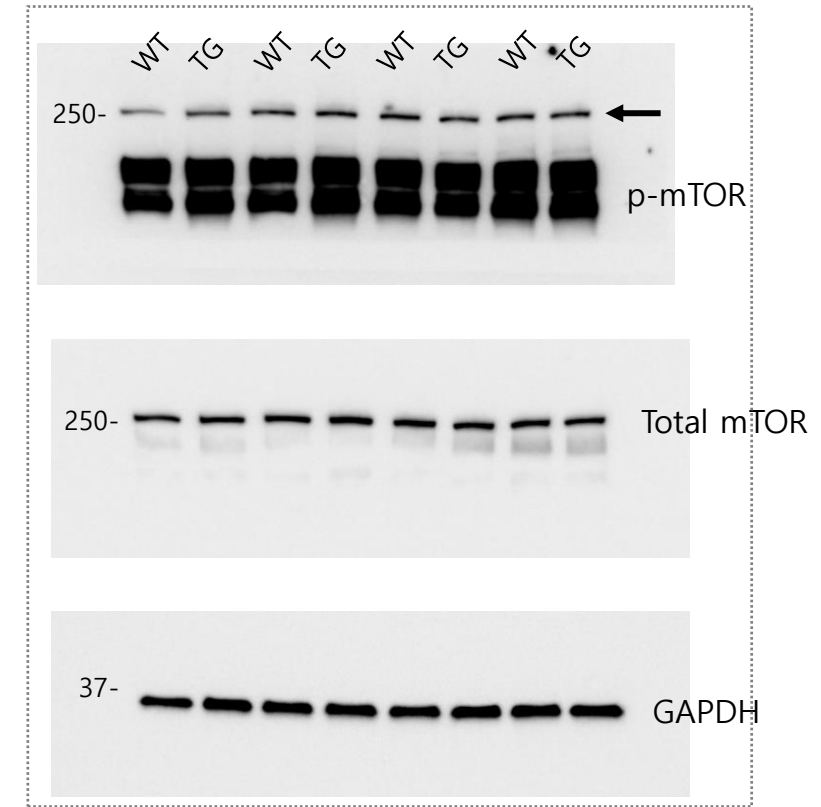

**Figure 1D.**

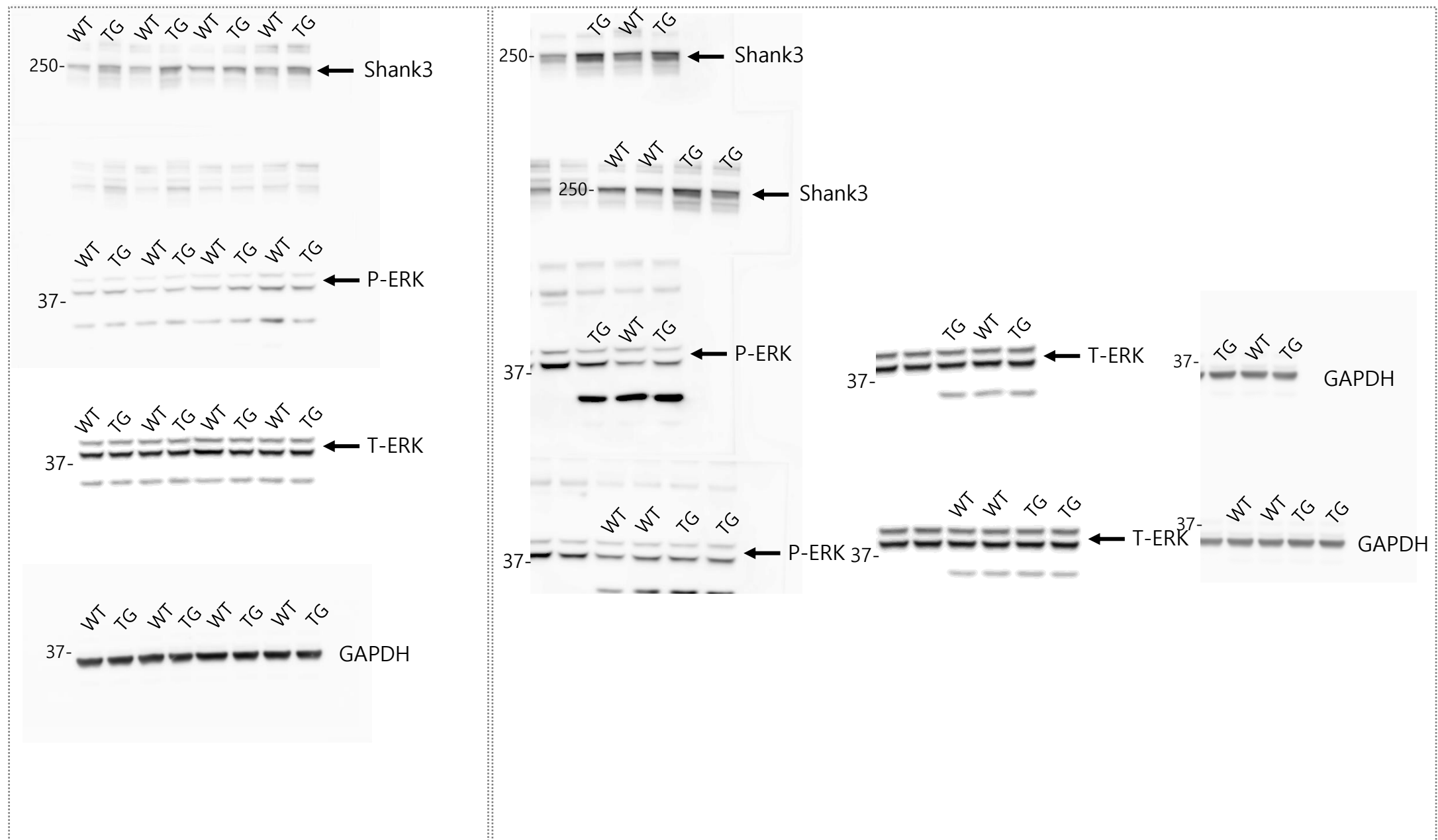

**Figure 1E.**

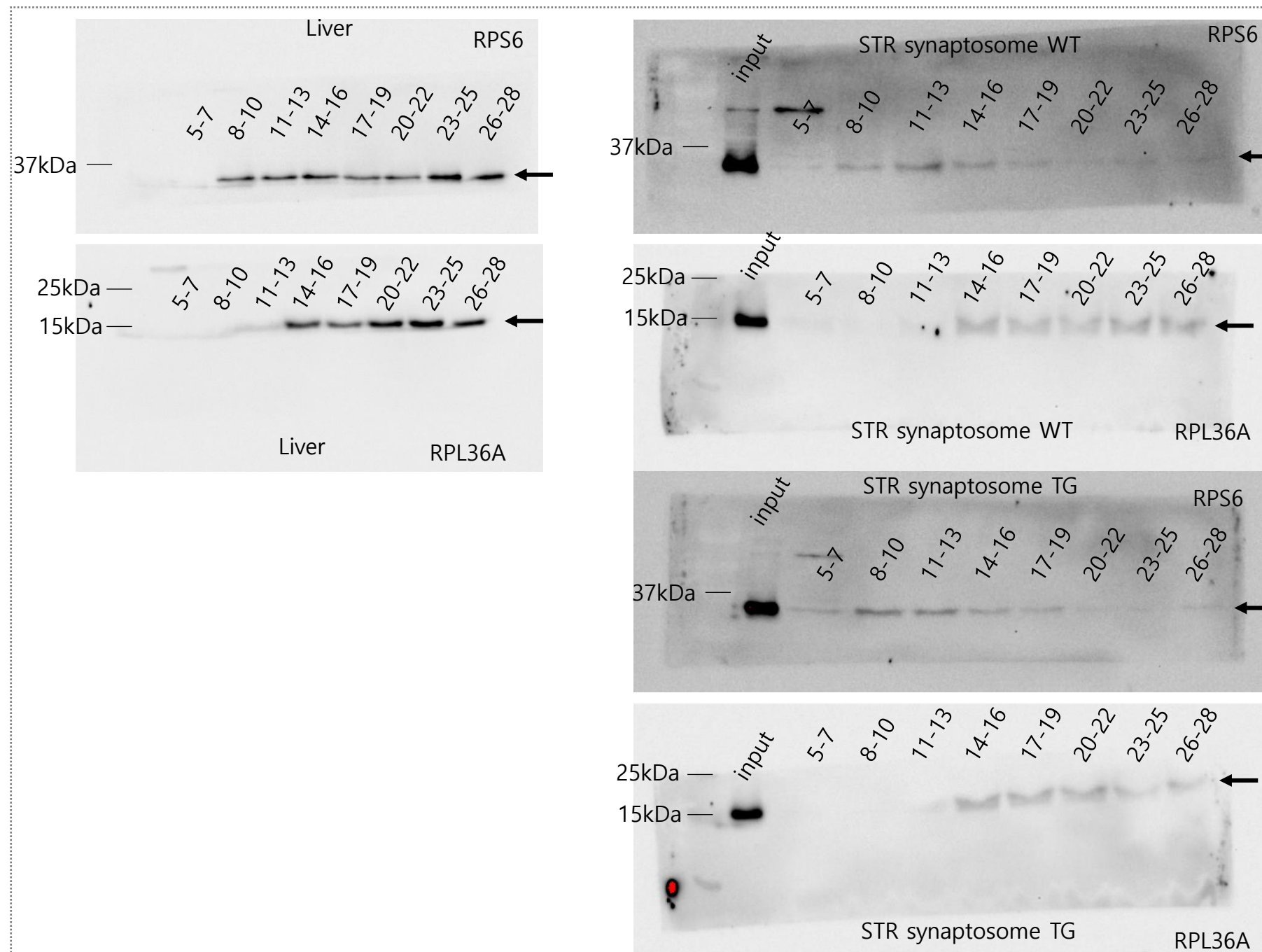

**Figure S3.**

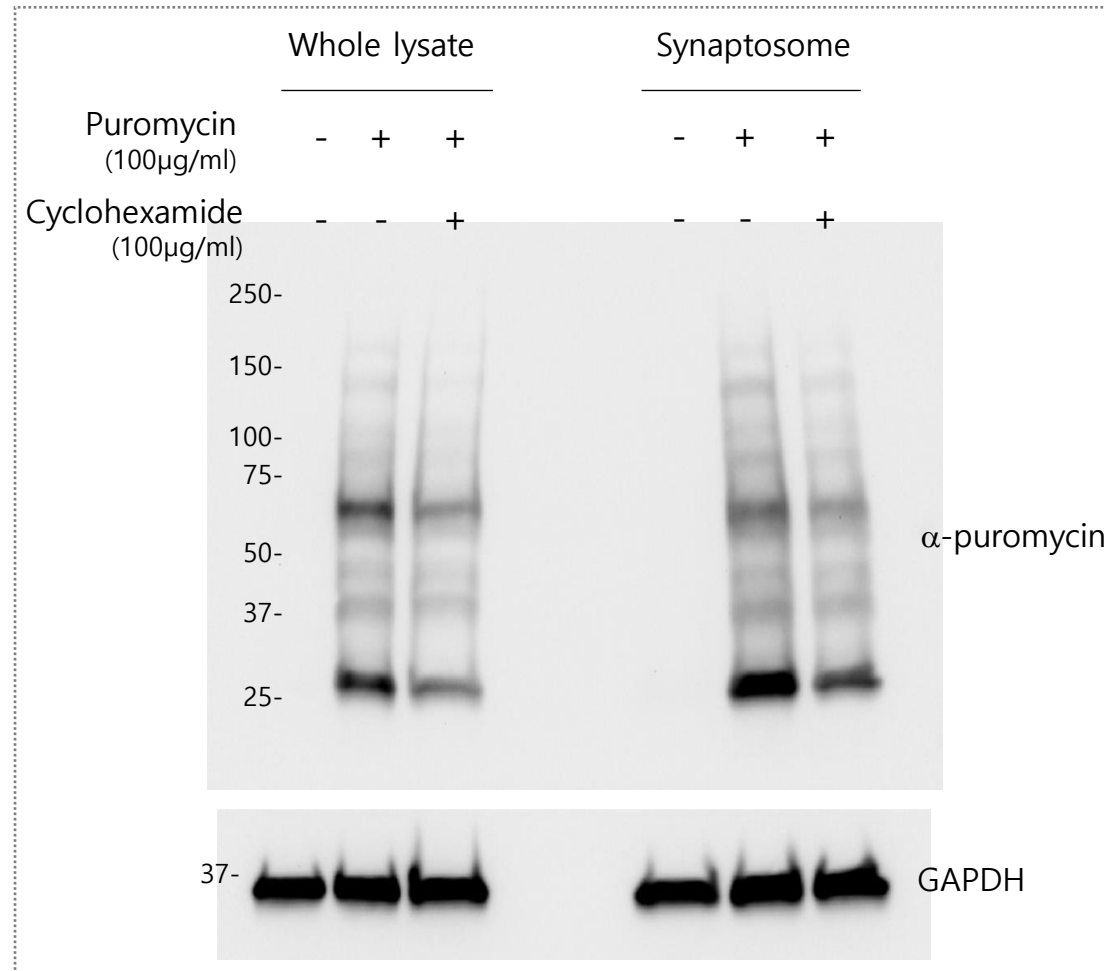

Supplement: Supplementary file 3 — Additional file 3. All raw images for entire membranes of Western blotting. [file 13041_2021_756_MOESM3_ESM.pdf]
